# Genomic characterization of putative allergen genes in peach/almond and their synteny with apple

**DOI:** 10.1186/1471-2164-9-543

**Published:** 2008-11-17

**Authors:** Lin Chen, Shuiming Zhang, Eudald Illa, Lijuan Song, Shandong Wu, Werner Howad, Pere Arús, Eric van de Weg, Kunsong Chen, Zhongshan Gao

**Affiliations:** 1 Institute of Fruit Science, The State Agriculture Ministry Laboratory of Horticultural Plant Growth, Development and Biotechnology, Zhejiang University, Hangzhou, 310029, PR China; 2 Allergy Research Center, Zhejiang University, Hangzhou, 310058, PR China; 3 IRTA. Centre de Recerca en Agrigenòmica CSIC-IRTA-UAB, Carretera de Cabrils Km2; 08348 Cabrils (Barcelona), Spain; 4 Plant Research International, Wageningen University and Research Centre, PO box 16, 6700AA, Wageningen, The Netherlands; 5 Key Laboratory of Pomology, Anhui Agricultural University, Hefei 230036, PR China

## Abstract

**Background:**

Fruits from several species of the Rosaceae family are reported to cause allergic reactions in certain populations. The allergens identified belong to mainly four protein families: pathogenesis related 10 proteins, thaumatin-like proteins, lipid transfer proteins and profilins. These families of putative allergen genes in apple (*Mal d 1 *to *4*) have been mapped on linkage maps and subsequent genetic study on allelic diversity and hypoallergenic traits has been carried out recently. In peach (*Prunus persica*), these allergen gene families are denoted as *Pru p 1 *to *4 *and for almond (*Prunus dulcis*)*Pru du 1 *to *4*. Genetic analysis using current molecular tools may be helpful to establish the cause of allergenicity differences observed among different peach cultivars. This study was to characterize putative peach allergen genes for their genomic sequences and linkage map positions, and to compare them with previously characterized homologous genes in apple (*Malus domestica*).

**Results:**

Eight *Pru p/du 1 *genes were identified, four of which were new. All the *Pru p/du 1 *genes were mapped in a single bin on the top of linkage group 1 (G1). Five *Pru p/du 2 *genes were mapped on four different linkage groups, two very similar *Pru p/du 2.01 *genes (*A *and *B*) were on G3, *Pru p/du 2.02 *on G7,*Pru p/du 2.03 *on G8 and *Pru p/du 2.04 *on G1. There were differences in the intron and exon structure in these *Pru p/du 2 *genes and in their amino acid composition. Three *Pru p/du 3 *genes (3.01–3.03) containing an intron and a mini exon of 10 nt were mapped in a cluster on G6. Two *Pru p/du 4 *genes (*Pru p/du 4.01 *and *4.02*) were located on G1 and G7, respectively. The *Pru p/du 1 *cluster on G1 aligned to the *Mal d 1 *clusters on LG16; *Pru p/du 2.01A *and *B *on G3 to *Mal d 2.01A *and *B *on LG9; the *Pru p/du 3 *cluster on G6 to *Mal d 3.01 *on LG12; *Pru p/du 4.01 *on G1 to *Mal d 4.03 *on LG2; and *Pru p/du 4.02 *on G7 to *Mal d 4.02 *on LG2.

**Conclusion:**

A total of 18 putative peach/almond allergen genes have been mapped on five linkage groups. Their positions confirm the high macro-synteny between peach/almond and apple. The insight gained will help to identify key genes causing differences in allergenicity among different cultivars of peach and other *Prunus *species.

## Background

In general, eating fruit is strongly recommended to improve health, but this can cause an allergic reaction in certain individuals. Most fruit crops in temperate and some subtropical zones belong to the Rosaceae family, mainly the subfamilies Maloideae and Prunoideae. Fruits from the Maloideae, like apple [1,2] and pear [3], and Prunoideae such as peach [4,5], sweet cherry [6,7], plum [8], apricot [9] and almond [10] have been reported to cause allergic reactions in Europe. The annual peach production in China has been around 6 million tons in recent years, and has lead to both an increase in peach consumption and more prevalent fruit allergy.

It is considered that general avoidance of fruit has a negative effect on health in allergic patients and also affects their quality of life. Therefore, the low allergic property of fruit is worth considering in new peach breeding programs. As cultivars of the same species differ in allergenicity [11], the selection and breeding of new, low-allergenic cultivars is becoming feasible by a multidisciplinary approach, such as in the EU-SAFE project [1], where doctors and plant geneticists identified the apple cultivar 'Santana' as low allergenic in the majority of Dutch patients with a history of allergy to apple [12]. A similar approach was initiated in peach in recent years through collaboration between China and Europe.

A variety of allergens from different fruits have been identified by means of experimental immunology and molecular biology, in particular by protein and gene identification and sequencing[13]. From the structural and biochemical point of view, fruit allergens identified belong to a limited number of protein families [13]. In the case of Rosaceae, the majority of allergens present in fresh fruit belong to four families: pathogenesis-related protein 10 (PR-10 protein, birch allergen Bet v 1 homologues), thaumatin-like proteins (TLP, PR-5 proteins), non-specific lipid transfer proteins (nsLTPs, PR-14 proteins) and profilins (PRF) [13]. Whole, unprocessed fruit containing PR-10 protein elicit allergy, while TLP, LTP and profilins are stable proteins because of intra-molecular disulfide bonds. These four allergen families in *Prunus *fruits are already on the international allergen list . In peach they are denoted as Pru p 1 (PR10), Pru p 2 (TLP), Pru p 3 (LTP), and Pru p 4 (profilin), and in almond as Pru du 1, 2, 3, and 4, respectively. Due to the common structure and properties of each protein family over a wide range of plant species, genera and even families, allergy cross-reactivity has been frequently observed, for example between Pru p 1 and Mal d 1 proteins from peach and apple respectively with Bet v 1 proteins in birch pollen [14]. Peach allergy occurs more often in southern Europe, and two different peach allergy profiles have been observed in Spain. One of these is the oral allergy syndrome (OAS) caused by Pru p1 and Pru p 4, the other is the systemic gastrointestinal symptom caused by Pru p 3 [15]. In China both profiles have also been reported after eating fresh peach [16]. In addition, some contact with peach fruit hair or even with pollen during the blooming season caused adverse reactions (from some patients' claims).

Genomic characterization of the sequences and linkage map positions of the four apple allergen gene families were reported in 2005 [17-19], and subsequent associations between the genetic constitution and allergenicity of different cultivars gave the first indications on the isoallergens (genes) and protein variants (alleles) involved in low and high allergenic responses  [20]. Comparison between apple (*Malus domestica*) and *Prunus *linkage maps suggests a high level of synteny of the linkage groups between these two genera [21]. For *Prunus *G1, the upper part contains several RFLP markers that aligned with apple LG13 and LG16 on which the majority of *Mal d 1 *genes are located, and the lower part aligned with LG8, where *Mal d 4.03A *is located. *Prunus *G3 aligns with apple LG9 where apple *Mal d 2.01A/B *and *Mal d 4.01 *were mapped. Therefore, some of the homologous allergen genes of *Prunus *fruit may be located at syntenic positions as compared with the apple genome.

In peach, differences in major allergen Pru p 1 (PR-10) and Pru p 3 (LTP) content among cultivars have been reported in recent years [11,22], which encouraged us to conduct research similar to that done in apple [17-19]. Advances in three aspects make the genomic research of *Prunus *allergen genes possible. First, a set of candidate genes is now available based on previous medical and molecular biology research [23-25]. Second, a large number of peach fruit ESTs are available in databases (mainly from researchers in Chile, Italy and USA), allowing us to find different members of the four putative allergen gene families. Third, a highly saturated genetic *Prunus *reference map T × E (based on the F2 progeny of 'Texas' almond and 'Earlygold' peach) and a simple bin-mapping strategy [26] provides an efficient and reliable way to map new genes.

## Results

PCR-based cloning and individual clone sequencing with genomic DNA templates of the *Prunus *T × E F1 hybrid (MB1-73, the plant used for the generation of the T × E F2 population by selfing), produced 35 consensus sequences (GenBank: EU424239-EU424273) represent 18 different gene loci. Within a single gene locus, two putative alleles from peach and almond were distinguished through marker test and sequence comparison. The total length of each sequence and length of its open reading frames (ORFs), exon and intron are summarized in Table 1. Primers used for linkage mapping are presented in Table 2. Each gene family is described below in more detail.

**Table 1:** Basic genomic features of four putative allergen genes identified from the MB1-73 plant (F1 hybrid between 'Texas' almond and 'Earlygold' peach)

**Genes**^1^	**Total length**^2^	**ORF**	**Exon 1**^2^	**Intron 1**^2^	**Exon 2**^2^	**Intron 2**^2^	**Exon 3**	**GenBank accessions**^3^	**Bin-map**^4^
*Pru p/du 1.01*	586	483	184	98	299			EU424239, EU424240	1:14
*Pru p/du 1.02*	772/762	483	184	233/223	299			EU424241, EU424242	1:14
*Pru p/du 1.03*	624	483	184	105/102	299			EU424243, EU424244	1:14
*Pru p/du 1.04*	615/622	480	184	124/131	296			EU424245, EU424246	1:14
*Pru p/du 1.05*	1503/1510	483	184	135	299			EU424247, EU424248	1:14
*Pru p/du 1.06A*	1871/2096	483	184	105/109	299			EU424251, EU424252	1:14
*Pru p/du 1.06B*	589	483	184	105	299			EU424249, EU424250	1:14
*Pru p 1.06C*	1432	483	184	109	299			EU424253	1:14
*Pru p/du 2.01A*	882/879	741	61	105/108	680			EU424256, EU424257	3:37
*Pru p/du 2.01B*	892	741	61	118	680			EU424258, EU424259	3:37
*Pru p/du 2.02*	1373/1271	741/729	52/58	118/180	689/671			EU424254, EU424255	7:25
*Pru p/du 2.03*	1296/1290	783/834	61	160	722/773			EU424260, EU424261	8:41
*Pru p/du 2.04*	1799/1825	993	61	110	694	678/704	238	EU424262, EU424263	1:50
*Pru p/du 3.01*	645	354	344	193	10			EU424264, EU424265	6:74
*Pru p/du 3.02*	514/513	372	362	98/97	10			EU424266, EU424267	6:74
*Pru p/du 3.03*	528/527	351	341	104/103	10			EU424268, EU424269	6:74
*Pru p/du 4.01*	1041	396	123	391	138	210	135	EU424270, EU424271	1:73
*Pru p/du 4.02*	754	396	123	209	138	131	135	EU424272, EU424273	7:56

^1 ^*p *and *du *represent peach (*P. persica*) and almond (*P. dulcis*), respectively.

^2 ^x/y indicates two different DNA lengths from 'Texas' and 'Earlygold' allele, the first is for 'Texas'.

^3 ^Two accessions are from alleles of 'Texas' almond and 'Earlygold' peach respectively.

^4 ^Bin map position is indicated by a first digit corresponding to the linkage group number followed by a colon and the distance in cM from the top of the linkage group to the last marker of this bin (i.e. 1:14 is linkage group 1 position 14 cM)

**Table 2:** Markers developed and cloning and sequencing primers for mapping

**Gene**	**Primer sequence**^1^	**Tm **°**C**^2^	**Cycles**^3^	**Allele length (nt)**^4^
*Pru du 1.01*	For-ATGGGTGTCTTCACATATGAGAG Rev-CTGACAACATATATACTGTAA**C**C	58	35	T-218
*Pru p 1.01*	For-TCATACAGCTACACCTTG**T**T Rev-CAATGAGCTTGAAGAGATTTG	58	35	E-205
*Pru p/du 1.02*	For-GTTGGAACCATCAAGAAGGAC ***GTTT***CATGAGCATACAGAACTATC	58	35	T-188 E-179
*Pru p/du 1.03*	For-GGTGAAGGTTAGCTAGTTGA Rev-***GTTT***CCATCCCTGTGCTTCACAA	58	35	T-153 E-150
*Pru p/du 1.04*	For-CTTTGGTGAAGGTAAGTTTAGA Rev-***GTTT***CATACCTGTAAGTGCTACCTGC	58	35	T-157 E-164
*Pru p/du 1.05*	For-GCTTCCAACATCAAGTAATACCT Rev-***G***TTTGGGACTAGGTTGTCAGCA	60	35	T-179 E-182
*Pru p/du 1.06A*	For-TAGTTATGAGTTGCTTGCAATGCT Rev-GAAAGTTCCAAAGTACATGTGC	58	35	1871, sequencing
*Pru du 1.06B*	For-ATCCCCAAGATTGATCCCC**T**G Rev-GATTAGAATTTAAGAGCTTACTGT	60/58	5/35	T-182
*Pru p 1.06B*	For-AAAGCCCTTGTTCTTGAAGCG Rev-GATTAGAATTTTAGAGCTTACT**C**C	60/58	5/35	E-212
*Pru p/du 1.06C*	For-CTAATTAATAACATAAATCTTGAAA**C**T Rev-TTGATCTCAACATCCCCTGC	56/54	5/35	E-272
*Pru du 2.01A*	For-CTTAGCATTCAACCAGCC**T**C Rev-GATGAGGTAGTCAGGGC**A**AG	60	35	T-175
*Pru p 2.01A*	For-CAATTGTCTCTGTAATCTGTTT Rev-ACGGAGCGTCCACAGAGTT	60	35	E-205
*Pru du 2.01B*	For-TTGCCCTGCCAATGTTAACGC Rev-GCACTGGGTCTTAAAGAAC**T**G	60	35	T-187
*Pru p 2.01B*	For-CAATTGTCTCTGTAATCTTTAG Rev-GGTCTTGAAGAGCTTAGAGTGA	60	35	E-622
*Pru p/du 2.02*	For-AAGAATCCATCAACTGAATCC Rev-CTACTAGGGTCTTCATCATCGG	56	40	T-271 E-315
*Pru p/du 2.03*	For-ACATGCGCCTCTGCGGACTA Rev-CCATTTCTTGCACGATTCAA	60	30	T-237 E-229
*Pru p/du 2.04*	For-TCAGTGTCAGGCTGTCAGTG Rev-CTCCAGATTCAGAGAGGTGC	60	35	T-267 E-235
*Pru p/du 3.01*	For-CACCATGCATACCCTACGTG Rev-CGTGAGGAATCCCTAAGTGG	60	35	530, sequencing
*Pru p/du 3.02*	For-CTTACCCCAATGCAAATGCT Rev-CCACTGAAACCAACAGCAGA	60	35	319, sequencing
*Pru p/du 3.03*	For-AGGCAGCCCTTACTTGTCCT Rev-CCACATGCTTCTCCGTATCA	60	35	413, sequencing
*Pru p/du 4.01*	For-AGAAGAAATCAGAAGCAACG Rev1-CAAGATACAACCCAGTTG**C**G Rev2-CTTTGTCCCACCAAGGAATAGA	55	35	T-276 E-631
*Pru du 4.02*	For-AAGTACATGGTCATCCAAG**C**C Rev-GTCCCTGAAATTGAAGTGTC	58	35	T-188
*Pru p 4.02*	For-GGCAACCACCTCTCT**C**CT Rev-CAAGATACAACCCAGTTG**C**G	55	35	E-363

^1^Designed mismatching nucleotides are in bold and underlined, added pigtail bases in bold and italics.

^2^, Annealing temperature (Tm)

^3^Number of PCR cycles. In the case of two values, a touch-down PCR was performed in two steps: the first number of the "Tm" and "Cycles" column refers to the Tm and number of cycles, respectively, of the first step.

^4^T and E refer to parental source from 'Texas' almond and 'Earlygold' peach, respectively. Genes mapped by direct sequencing are indicated by 'sequencing'

### *Pru p/du 1 *(PR-10) genes

**Figure 1:**
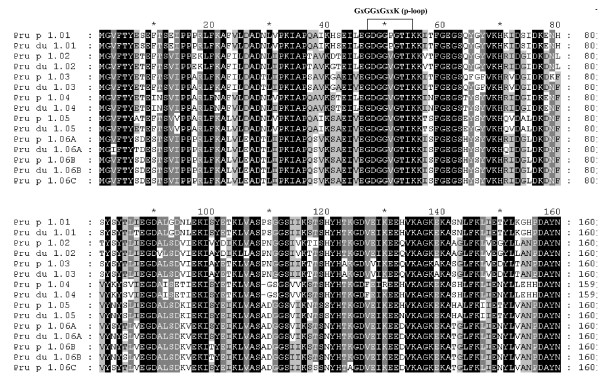
**Alignment of amino acid sequences of Pru p/du 1 members identified in the *Prunus *'Texas' almond × 'Earlygold' peach plant MB1-73.** p, peach, du, almond. Four levels of identity are shown: (1) black, 100%; (2) grey, 80%; (3) light grey, 60%; and (4) white.

**Figure 2:**
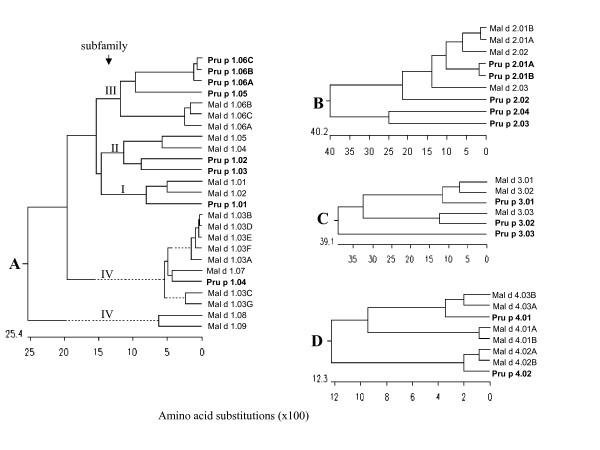
**Phylogenetic trees derived from the deduced PR-10, TLP, LTP and profilin (PRF) families in peach (Pru p 1–4) and apple (Mal d 1–4) based on our genomic sequences and ESTs (in bold) retrieved from the DNA NCBI Database (see their GenBank accession numbers below).** A: PR-10 family. Peach: Pru p 1.01 [GenBank:EU424240], Pru p 1.02 [GenBank:EU424242], Pru p 1.03 [GenBank:EU424244], Pru p 1.04 [GenBank:EU424246], Pru p 1.05 [GenBank:EU424248], Pru p 1.06A [GenBank:EU424252], Pru p 1.06B [GenBank:EU424250], Pru p 1.06C [GenBank:EU424253]. Apple: Mal d 1.01 [GenBank:AY789238]; Mal d 1.02 [GenBank:AY789241]; Mal d 1.03A [GenBank:AY789263]; Mal d 1.03B [GenBank:AY789265]; Mal d 1.03C [GenBank:AY789266]: Mal d 1.03D [GenBank:AY789267], Mal d 1.03E [GenBank:AY789269], Mal d 1.03F [GenBank:AY789271], Mal d 1.03G [AY789274], Mal d 1.04 [GenBank:AY789244], Mal d 1.05 [GenBank:AY789246], Mal d 1.06A [GenBank:AY789249], Mal d 1.06B [GenBank:AY789251], Mal d 1.06C [GenBank:AY789254], Mal d 1.07 [GenBank:AY789257], Mal d 1.08 [GenBank:AY789260], Mal d 1.09 [GenBank:AY789262]. B: TLP family. Peach: Pru p 2.01A [GenBank:EU424257], Pru p 2.01B [GenBank:EU424259], Pru p 2.02 [GenBank:EU424255], Pru p 2.03 [GenBank:EU424261], Pru p 2.04 [GenBank:EU424263]. Apple: Mal d 2.01A [GenBank:AY792599], Mal d 2.01B [GenBank:AY792603]; Mal d 2.02 (consensus sequence derived from ESTs, [GenBank:CO9044477, CV082311, CO723595, CN445021] and Mal d 2.03 (consensus of 8 ESTs: [GenBank:CO866703, CO901275, CN495042, CV084040, CO866711, CN444138, CO866347, CN491711] [18]. C: LTP family: Peach: Pru p 3.01 [GenBank:EU424265], Pru p 3.02 [GenBank:EU424267], Pru p 3.03 [GenBank:EU424269]; Apple: Mal d 3.01 [GenBank:AY572501], Mal d 3.02 [GenBank:AY572517], Mal d 3.03 [GenBank:DY256246]. D: Profilin Family: Peach: Pru p 4.01 [GenBank:EU424271], Pru p 4.02 [GenBank:EU424273]; Apple: Mal d 4.01 [GenBank:AY792608], Mal d 4.02A [GenBank:AY792610], Mal d 4.02B (consensus of 3 ESTs, [GenBank:CN578860, CN992900, CV082623], Mal d 4.03A [GenBank:AY792616], Mal d 4.03B (consensus of 3 ESTs, [GenBank:CO722645, CO052497, CO418275].

**Figure 3:**
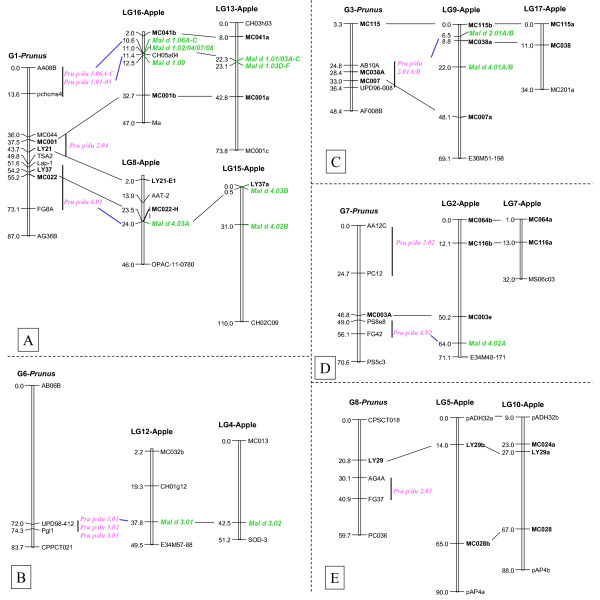
**Linkage map positions of the four allergen gene families in *Prunus *(T × E) reference map and alignment with those in apple (*Malus domestica*) reference map**. Relevant linkage map information was derived from *Prunus *T × E updated maps (Arús et al.) and apple (Maliepaard et al., TAG, 1998, 97:60–73; Gao et al 2005, TAG 110:479–491; 111:174–183 and 1087–1097). An initial reference for alignment is based on Dirlewanger et al. (PNAS, 2004, 101:9891–9896). A bar indicates map position range of a bin. Homologous allergen genes and common RFLP probes are underlined to show their synteny.

According to four Pru p 1 cDNA reference sequences derived from peach fruit in the GenBank database, we obtained 15 different PR-10 DNA sequences [GenBank: EU424239-EU424253] from MB1-73 by PCR cloning, and classified them into eight distinct gene members based on their DNA sequence identity less than 98%, of which four were new (*Pru p/du 1.05 *and *Pru p/du 1.06A*-*C*) (Table 1) and formed a new group distinct from the initial reference sequence [GenBank:DQ251187] (*Pru p 1.01*). All *Pru p/du 1 *genes had a single intron with variable size, (98–233 nt) and two exons of 184 nt and 299 nt each, except in the second exon in *Pru p/du 1.04 *that was 296 nt long. Alleles derived from almond cv 'Texas' and peach cv 'Earlygold' had different intron sizes in four *Pru p/du 1 *members (Table 1), used to map these genes in polyacrylamide gel electrophoresis (PAGE). DNA sequence divergence between allele from peach and almond is very low (1%–2.1%). Amino acid (aa) sequences from these genes were deduced from their coding DNA sequences (Figure 1). Most Pru p/du 1 had 160 aa, except for Pru p/du 1.04 with 159aa, with the common feature of a p-loop (GxGGxGxxK). Overall aa sequence identity among different isoallergen was 71.9%–98.8%, and three Pru p 1.06 members (A, B, C) have about 98% aa sequence identity (Figure 1). Segments of identical aa sequence were found in several locations, especially surrounding the p-loop. Pru p/du 1.01 had a proline (P) residue in the p-loop, which is similar to Mal d 1.01 and 02 and all Bet v 1, while the other Pru p/du 1 isoallergens had a valine (V) in this position. The predicted protein molecular weights were in the range of 17.1–17.5 kDa, and pI values ranged from 4.9 to 6.0. Comparing the aa composition between alleles from peach and almond of each gene in Figure 1, we found that there was only one difference for Pru p/du 1.01, 02, 03, 04 and 06B, while Pru p/du 1.05 and Pru p/du 1.06A differed by 2 and 5 aa, respectively. A phylogenetic tree of the deduced amino acids from peach cv. 'Earlygold' alleles and those in apple (mainly cv. 'Fiesta') (Figure 2A) showed that at least one Pru p 1 isoallergen member is present in each of the four subfamilies of apple *Mal d 1 *genes (PR-10) identified in a previous study [17]. Furthermore, we can see in the subfamily III branch that Pru p 1.05 and three highly similar Pru p 1.06 members (A-C) clustered with three Mal d 1.06 members (A-C), while Pru-1.04 clustered together with Mal d 1.07, one of the apple intronless genes in subfamily IV. All eight *Pru p/du 1 *genes mapped to bin 1:14, at the top of G1 (Figure 3A, Table 1).

### *Pru p/du 2 *(TLP)

**Figure 4:**
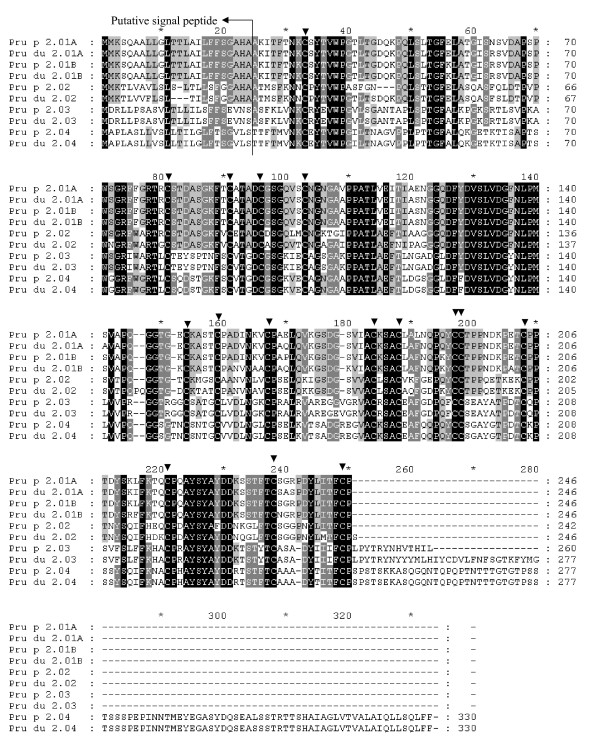
**Alignment of amino acid sequences of Pru p/du 2 members identified in the *Prunus *'Texas' almond × 'Earlygold' peach hybrid MB1-73**. Four levels of identity are shown: (1) black, 100%; (2) grey, 80%; (3) light grey, 60%; and (4) white. The signal peptides were deducted by comparison with other TLPs in Rosaceae fruits or other plants. 16 conserved cysteine residues are arrowed.

Analysis of the peach TLP sequences in the NCBI identified four members of this gene family at the start. A single primer pair based on [GenBank:AF362988] resulted in four very similar sequences assigned as two separate genes (*Pru p/du 2.01A *and *2.01B*) with minor intron size differences. Genome walking to upstream was employed in *Pru p/du 2.02 *to search for more polymorphisms and downstream to *Pru p/du 2.03*, since its initial reference sequence was partial. Five members of *Prunus *TLPs were identified after analysis of the genomic sequences [GenBank: EU424254-EU424263]: *Pru p/du 2.01A *and *-01B*, *Pru p/du 2.02*, -*03 *and -*04 *(Table 1), of which *Pru p/du 2.01B *is new. The *Pru p/du 2 *members had distinct genomic structure (Table 1). Three of them had one intron separating a short (58 or 61 nt) and long (671–773 nt) exon, while one member (*Pru p/du 2.04*) had two introns. Comparing the deduced precursor of *Prunus *TLPs with the reference TLP amino acid sequences in the Rosaceae and other plants, we assigned the putative signal peptides to deduce the mature protein (Figure 4). Pru p/du 2.02 had a shorter signal peptide, 23 aa in peach and 21 aa in almond, while the other four members had a 24 aa signal peptide. They differed considerably in protein size, with Pru p/du 2.03 and Pru p/du 2.04 having longer peptides in the C-terminal than Pru p/du 2.01 and 02. Amino acid sequences identity from alleles from peach and almond for five Pru p/du 2 members are different, they are 84.4% for Pru p/du 2.02, about 96% for Pru p/du 2.01A and -01B, 98% for Pru p/du 2.03, and 99.4% for Pru p 2.04 p/du 2.04.

Based on the percentage amino acid identity among five Pru p 2 isoallergens, Pru p 2.01A, 01B and 2.02 were clustered with four apple Mal d 2 (01A, 01B, 02 and 03) (Figure 2B) proteins. Pru p 2.01A and 2.01B had 96.7% identity in their aa sequence, their closest homologous members being apple Mal d 2.01A and B. Pru p 2.03 and 2.04 are quite distant from 2.01 and 2.02 (Figure 2B) and no close counterparts were found in apple. The overall sequence identity level in amino acids among the four Pru p 2 proteins was around 40% (Figure 2B), but a long sequence region (DFYDVSLVDG [F/Y]NLPM) around position 130, and the typical 16 cysteine (C) to form 8 disulfide bonds, were conserved in all Pru p/du 2 members. Mature Pru p 2.01A, 02 and 04 proteins were acidic (pI around 4–5), whereas Pru p 2.01B and 3 were basic (pI 7.9–8.17). Mature Pru p 2.01 and 02 had a molecular weight of about 23 kDa. Mature Pru p 2.03 differed in length and weight with mature Pru du 2.03, with the molecular weight being 25.23 kDa and 27.46 kDa respectively. Mature Pru 2.04 had the highest number of aa (307) with a calculated molecular weight of 30.7 kDa. Unlike the *Pru p 1 *genes that were placed in a single bin, these five *Pru p/du 2 *members mapped on four linkage groups: *Pru p/du 2.01 *A and B on G3, *-2.02 *on G7, *-2.03 *on G8, and *-2.04 *on G1 (Figure 3A, C, D, E).

### *Pru p/du 3 *(nsLTP)

Four peach and almond nsLTP members were traced from the NCBI DNA database, three of them have complete coding sequences, the fourth (GenBank:BQ641139) is a partial almond cDNA sequence distant from the other three. Genomic cloning and sequencing was done with the first three members *Pru p/du 3.01*-*03 *[GenBank: EU424264-EU424269]. *Pru p/du 3 *gene sequences have two exons and one intron (Table 1). The size of the second exon was conserved and extremely short (10 nt), while the first was variable (344, 362 and 341 nt). Sequences from peach and almond LTPs are highly conserved in exon and intron size, with only one base pair length difference in introns of *Pru p/du 3.02 *and *3.03 *(Table 1).

**Figure 5:**
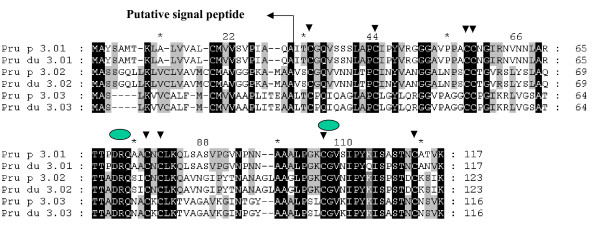
**Alignment of amino acid sequences of LTPs members identified in the *Prunus *'Texas' almond × 'Earlygold' peach hybrid MB1-73.** Eight conserved cysteine residues are arrowed. The lipid-binding motifs are shown by green oval.

The deduced pre-mature Pru p 3 proteins had different signal peptide lengths of 26 aa, 30 aa and 25 aa for Pru p/du 3.01–03, respectively (Figure 5). In peach and almond (E and T), Pru p/du 3.01 alleles had identical signal amino acids, but their mature proteins differed in 5 aa, of which 4 are at the C-terminal end. Pru p/du 3.02 had identical aa, and Pru p/du 3.03, only one amino acid difference P/T at position 88. The three *Prunus *LTPs isoallergens differed greatly in their signal peptide region, with about 40–80% sequence identity (Figure 2C), their alignments were according to the eight conserved cysteine residues (Figure 5). All mature Pru p/du 3 were alkaline (pI = 8.5–9.22) and their molecular weight was around 9 kDa. Pru p 3.01 grouped closely with apple Mal d 3.01 and 02 (Figure 2C), and Pru p 3.02 with the putative Mal d 3.03 (e-cloning analysis assigned). Pru p 3.03 is distant from Pru p 3.01 and -02, and shares about 50% aa identity with the others. Interestingly, all three *Pru p 3 *genes mapped on bin 6:74 (Figure 3B). One of them, *Pru p 3.03 *(as *Ltp2*), had previously been mapped using the whole TxE population and was located at 72.5 cM from the top of G6 [19].

### *Pru p/du 4 *(profilin, PRF)

**Figure 6:**
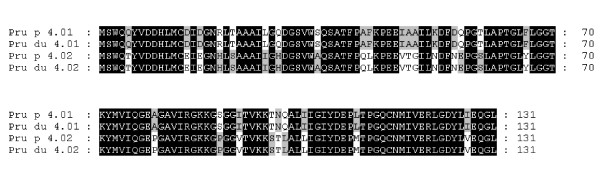
Alignment of amino acid sequences of two profilin members from peach and almond.

Genomic sequences for two profilin genes *Pru p/du 4.01 *and *Pru p/du 4.02 *were obtained based on their cDNA data [GenBank: EU424270-EU424273]. Both genes have three exons with conserved length, 123, 138 and 135 nt, in agreement with that found in most plant species. DNA polymorphisms in *Pru p/du 4.01 *occurred mainly in the first intron, but in *Pru p/du 4.02 *they were in the first and second exon. These two genes gave the same protein sequences for peach and almond (Figure 6), although a few synonymous polymorphisms existed at the gDNA level. Molecular weights were 14.06 and 14.19 kDa for Pru p 4.01 and 4.02, respectively, and both were acidic (calculated pI 4.5 and 4.6). Compared with the six known Mal d 4 isoallergens, Pru p 4.01 was similar to Mal d 4.03A and B (93.1% and 93.9% identity), while Pru p 4.02 was similar to Mal d 4.02A and B (95.4% and 96.9% identity) (Figure 2D). *Pru p 4.01 *was mapped on G1 and *Pru p 4.02 *on G7 (Figure 3A and 3D).

## Discussion

This study provides basic genomic sequences and map positions of four allergen families in *Prunus persica *(peach) and *Prunus dulcis *(almond). This information is a prerequisite for unravelling the genetic basis of allergenic characteristics of *Prunus *fruits, and is likely to be similar for other *Prunus *species due to the high level of synteny of the species within this genus [21].

### Gene families and relevance of individual genes in allergenicity

Gene family is becoming a prevailing topic in genomic research. It is thought that the pressure to conserve protein sequence and structure is associated with an increase in copy number of certain genes during evolution [27]. Although food allergens belong to a limited number of protein families [28], each protein family in a single species can be putatively encoded (experimentally demonstrated in some cases) by multiple genes in specific tissues, thus making the in vivo allergic reaction very complex. There is a need to identify genes implicated in the allergic reaction from the many putative allergen genes existing. Searching the updated *Arabidopsis thaliana *DNA sequence database, we found 29 putative *Pru p 1*-homologous genes scattered over the five chromosomes (Table 3). More than half of these (17/29) are located on chromosome 1 in four clusters, each with 3 to 7 genes. Nineteen TLP genes are present on four chromosomes. LTP genes form a large gene family with 98 members, more or less equally distributed over all five chromosomes, and profilin is a small gene family with 5 members mapping to three chromosomes. It is probable that, in peach and apple, only a small fraction of PR-10, TLP and LTP gene members are relevant to allergenic trait.

**Table 3:** Members of four gene families related to allergenicity, pathogenesis related 10 proteins (PR-10), thaumatin-like proteins (TLP), lipid transfer proteins (LTP) and profilin (PRF) on the five *Arabidopsis thaliana *chromosomes traced from the GenBank database

**Gene**	**Chrom-1**	**Chrom-2**	**Chrom-3**	**Chrom-4**	**Chrom-5**	**Total**
***PR-10 ***	17	3	2	3	4	29
***TLP ***	8	2		6	3	19
***LTP***	19	14	16	25	24	98
***PRF***		2		2	1	5

A limited number of putative allergen genes may be expressed in consumed fruits, their expression profiles at mRNA and protein level are essential to allergenicity. Evidence of cDNA present in the ripe fruit is not sufficient. The need to qualify and quantify their individual or combined allergenicity is a major challenge for further investigation, where the application of new experimental and computational tools in genomics and proteomics will be crucial. Results in this area are starting to emerge and integrate, such as differential expression of LTP 1 (Pru p 3.01) and LTP2 (Pru p 3.02) [29], the expression studies of Pru p 3.01 (Pp-LTP1 in two peach genotypes [23], where major differences were detected in expression at mRNA and protein accumulation levels between them, and the research on IgE binding capacity [30], where specific IgE-binding protein regions of Pru p 3 were found for two types of allergy (OAS and systemic) by the mimotope mapping technique. Of four Pru p 2 isoallergen genes, a clone PpAz44 [GenBank: AF362988] matching to *Pru p 2.01 *was abundantly expressed in ripe peach fruit, while clone PpAz8 [GenBank:AF362987] (Pru p 2.02) was undetected by northern blot analysis [24]. *Pru p 2.03 *and *Pru 2.04 *were identified from the fruit ESTs, the latter may be in large amount of transcript, since 29 ESTs can be assembled into one consensus gene. A very low level of Pru p 4 (profilin) was has been identified as pollen-associated allergen, and two isoallergen genes were isolated from fruit tissue [25]. Different *Pru p 1 *genes expression was not reported previously, our preliminary expression study was performed by means of real-time PCR for six Pru p 1 isoallergen genes on full ripe fruits of two cultivars. The result showed that the most abundantly expressed member is *Pru p 1.01*, followed by *Pru p 1.06*, then very low expression level for *Pru p 1.04 *and *-1.05*, while *Pru p 1.02 *and *-03 *were hardly detected (data not shown).

In apple, a very recent comprehensive gene expression of four classes of putative allergens in apple fruit has gained insight of the genetic and environmental factors affecting the allergenic potential of apples [31]. They showed that some putative members are statistically different in transcript accumulation in ripe fruit, while others were not detectable. This was a very nice reference to think about comparative transcription and protein expression for all set of peach putative allergen genes among diverse peach cultivars.

We found that the putative genes encoding the major allergens reported in peach fruit, Pru p 1 and Pru p 3 [15], were located on two linkage groups, G1 and G6. Based on their bin map positions, we can use SSR markers tightly linked or flanking these two major *Prunus *allergen genes for a diversity survey of a large number of genotypes to search for potential candidates for further sequencing of the allergen genes. Clinical evaluation of allergenicity for different peach cultivars is needed for association analysis to find low allergenic alleles.

### Comparative mapping between *Prunus *and *Malus *using the sequence and position of allergen genes

Dirlewanger et al. [21] compared the *Prunus *and apple genomes using a limited number of common markers. They found that three *Prunus *linkage groups (G1, G3 and G4) were syntenic to seven apple linkage groups, G1 to LG13 and LG16 at the top and LG8 at the bottom, G3 to LG9 and LG17, and G4 to LG5 and LG10. Information is also available on the correspondence between homoeologous linkage groups within the apple genome obtained initially with RFLPs by Maliepaard et al. [32], to which various allergen genes and SSRs were recently added by Gao et al. [17-19] and Celton et al. [33], respectively. With the availability of genomic sequences and the linkage map positions of the four allergen-families in apple (26 apple allergen genes on eight linkage groups) [17-19] and peach (18 genes on 5 linkage groups, this study), we have additional information useful for updating the synteny between these two species.

Three of the eight *Prunus *bins where we located putative allergen genes corresponded to known syntenic and colinear regions of the apple genome (Figure 3): the *Pru p/du 1 *on G1, corresponding to regions with clusters of homologous genes (*Mal d 1*) of apple on LG13 and LG16, *Pru p/du 4.01 *on the lower part of G1 and *Mal d 4.03 *on LG8, and *Pru p/du 2.01A/B *on G3 and *Mal d 2.01A/B *on LG9. Three more syntenic regions were detected in this study: *Pru p/du 4.02 *on G7 and the region of LG2 where *Mal d 4.02 *maps, *Pru p/du 3.01*-*03 *on G6 corresponding to *Mal d 3.01 *on LG12 and *Mal d 3.02 *on LG4; and *Pru p/du 4.01 *which, in addition to the region of LG8 already mentioned, is syntenic with the upper part of LG15 where *Mal d 4.03B *maps. No counterparts were found to the *Pru p/du 2.02, -03*, and *-04 *genes, which were mapped on G7, G8 and G1, respectively, on the apple genome. The positions of these three genes in the *Prunus *genome indicate syntenic regions of apple where additional *Mal d 2 *genes may be located. Because *Mal d 1 *on apple LG 13 and LG16 are clustered in a region of about 2 cM, so we can deduce our mapped *Pru p 1 *genes are also in a cluster.

There are two hypotheses on the origin of the two genomes that generated the apple amphydiploid genome (*2n *= 34). One proposes that these were from members of two Rosaceae subfamilies, one from the Amygdaloideae (thus closer to *Prunus *with *x = 8*) and another from the Spiraeoideae (*x = 9*) [34]. The other hypothesis proposes that both ancestral genomes had a basic chromosome number of *x = 9 *at least one being Spiraeoideae (*x = 9*) [35]. Combining map position and sequence comparison, the cluster of *Pru p 1 *genes mapped on *Prunus *G1 is more similar to that of the *Mal d 1 *genes of LG16 than to LG13: a) Mal d 1 isoallergen genes were classified into four subfamilies and members of sub-families were distributed asymmetrically to LG 13 and LG16 (Figures 2, 3), i.e. LG13 contains Mal d 1 members from subfamilies I and IV, but LG16 contains representative members of the four subfamilies; b) *Pru p 1 *genes fall into the four subfamilies as defined in apple, and had a higher deduced amino acid sequence similarity to the *Mal d 1 *members on LG16 (Figure 2 and data of percentage amino acid identity in the Results section). These results favor the two primitive genomes for apple and one is closer to *Prunus *than the other.

### Bin mapping feasibility and effectiveness

The bin mapping approach provides a fast and cheap way to map new genes [26] that has worked very well in our hands. Once we cloned and sequenced the two putative alleles for each gene from MB1-73, we either created an allele-specific marker and genotyped the eight plants of the bin set, or cloned the fragments from the individuals of the bin set and sequenced them to determine their genotypes. One of the advantages of using the T × E population for bin mapping is that, due to its inter-specific nature, polymorphism is very high [26]. The disadvantage of the bin mapping approach is that there is a loss of precision in marker location (8–25 cM), but this can be solved for markers of particular interest by running the whole population to establish a more precise position or narrow down a region where closely related genes or common RFLP markers in apple has been fine mapped. For example, we estimate *Pru p/du 1 *genes (in bin 1:14) are in a cluster and likely around 13 cM from the top based on location of *Mal d 1 *genes and anchor marker MC001 (Figure 3A). A very large number of ESTs from peach fruit are on public databases. This information is essential to find candidate genes to be mapped, and their positions compared to that of major genes or quantitative trait loci of interest mapped using phenotypic data. Bin mapping, as in this case, can facilitate the positioning of these candidate genes. Exchange of DNA and genotype data of the T × E bin set in the peach research community is helping candidate gene mapping, with genetic analysis and breeding applications following on.

## Conclusion

Eighteen putative *Prunus *allergen genes were located on five of the eight linkage groups of this genus. Genomic characterization of *Prunus *allergens provided further evidence for synteny between *Prunus *and *Malus*. This information will be directed towards identifying the key genes or isoforms causing the differences in allergenicity among different cultivars by additional gene and protein expression and genetic association studies. These results are a first step towards the understanding of allergenicity caused by peach fruit and for the development of new cultivars with enhanced health properties and subject to specific recommendations on fruit consumption.

## Methods

### Plant materials

PCR cloning and sequencing for targeting allergen genes to identify different gene members and alleles was mainly on the 'Texas' (T) almond and 'Earlygold' (E) peach hybrid (MB1-73) used to generate the reference mapping population (T × E) and it is now used by the international *Prunus *community. Therefore, two putative alleles, one from 'Texas' (T) and another from 'Earlygold' (E), will be simultaneously amplified with a pair of primers based on a reference sequence. For T and E allele-specific marker tests, DNA from T and E were used as controls. Eight plants were used for the bin set (the set of plants used for bin mapping): the grandparent E, the parent MB1-73, and six plants from the F2 progeny (# 5, 12, 23, 30, 34, 83) [26]. DNA samples were isolated and provided by IRTA using standard protocols [36].

### PCR primers, cloning and sequencing

Four families of putative allergens from peach and almond, PR-10 (Bet v 1 homologous gene), TLPs, LTPs and profilins (PRF), were searched in the GenBank, most of them were derived from peach fruit tissues. The sequences obtained were assembled using Seqman program (DNAstar, Lasergene 6) to identify different gene members for each family. DNA sequences were assumed to belong to different gene members when they displayed less than 98% identity. This resulted in four members for *Pru p1 *(PR10) and *Pru p 2 *(TLP), three for *Pru p 3 *(LTP) and two members for *Pru p 4 *(profilin). Primer pairs were designed using the software program PRIMER DESIGNER v. 2.0 (Scientific and Educational Software, Cary, N.C.) based on these sequences. Preferably, the forward and reverse primers covered the whole region of the gene encoding sequences (Table 4). Detailed PCR cloning and sequencing procedures have been described previously [19], here we mention only the key points and changes. First, the melting temperature for amplification was optimized by gradient PCR with hybrid (MB1-73) DNA, then PCR was performed in two steps with *Pfu *polymerase (Stratagene, La Jolla, Calif.) and *Taq *(Takara Biotechnology, Dalian, China) using an Eppendorf Masterycler. The amplified fragments were purified by Qiaquick Gel Extraction Kit (Qiagen, Germany), ligated into the pGEM-T easy vector (Promega, Madison, Wis.) and used to transform DH5α competent cells (Takara Biotechnology, Dalian, China) according to the manufacturer's protocol. For each purified fragment, 6–8 white colonies were selected for plasmid isolation and sequencing by Invitrogen (Shanghai, China) using an ABI 3730 (Applied Biosystems, Foster City, Calif.) platform. If there are more than two distinct sequences with initial one primer pair, additional colonies were sequenced to explore new gene members. All primers were synthesised by Invitrogen (Shanghai, China).

**Table 4:** PCR primer pairs used for genomic cloning of the different peach/almond allergen genes and for (bin) mapping the genes

**Primers group**	**Reference Sequence **^1^	**Primer name**	**Primer sequence (5' – 3')**	**Tm °C/cycles**		**Product**	**Gene **
				
				***Pfu***	***Taq***	**(nt)**	
1	DQ251187	PpPr10f PpPr10r	ACCATGGGTGTCTTCACATA AATTTAGTTGTAGGCATCGG	56/30	58/2	586/623	Pru p/du 1.01/05
2	DY646736	Pp1-2f Pp1-2r	GTTTGTCCTTTAAACTCTCC GATTTAGTTGTAGGCATCGG	53/30	55/2	762/765	Pru p/du 1.02
3	DY647300	Pp1-3f Pp1-3r	CTTTGATCAGTTTCCCAAGT TTAGTTGTAGGCATCAGGGT	53/30	55/2	624	Pru p/du 1.03
4	DY653062	Pp1-4f1 Pp1-4r	TTTACAGAATCATGGGTGTG TTAGTTGTAGGCATCATGGT	53/30	55/2	615/622	Pru p/du 1.04
5	GW1	Pp1-1A-GSP1 Pp1-1A-GSP2	AACCTTCACCAAAGCTAGTCTTCTTGATG GTCAGCATCCAGAACAAGGGCTTTGAACA			1100	Pru p/du 1.05 /6A/C
6	GW2	P1-1A3-GSP1 P1-1A3-GSP2	GGTGTCTTCACATACTCAGACGAGTC CCACCTCAGTCATCCCCCCACCAAGA			1546	Pru p/du 1.06A/C
7		P1-1-6 PpPr10r	CACCTCCAGTGCTCATAGC See above	57/30	59/2	720	Pru p/du 1.05
8		P1-7f P1-7r	ATGGGTGTCTTCACATACTCAG TTTAGTTGTAGGCATCTGGGT	57/30	59/2	593	Pru p/du 1.06A/B
9		P1-8f P1-8r2	TAGTTATGAGTTGCTTGCAATGCT GGGCATGCTTGCTTTGGTAACTC	58/30	60/2	1346	Pru p/du 1.06C
10		P1-8f P1-9r	See above GAAAGTTCCAAAGTACATGTGC	58/30	60/2	1908	Pru p/du 1.06A
11	AF362988	PpTL2f PpTL2r	ACAGCAAGCCAATTAAGACA GCTTATGGGCAGAATGTGATGAG	56/30	58/2	879/892	Pru p/du 2.01A/B
12	AF362987	PpTL1f PpTL1r	ATGATGAAGACCCTAGGAGCAG GTCGAGAGTCCTATCTTTAT	56/30	58/2	974	Pru p/du 2.02
13	GW3	PTLP1-GSP1 PTLP1-GSP2	GATAATTGAGGATTTCCAAAGGAGGCTG AGGAGGGTTAAGCTGAGGCTGAGGACTG			500/1500	Pru p/du 2.02
14	BF717226, DW349103	PpTLP3f PpTLP3r	AAGTAAAGTTCTTTGGCGTC CACGTGGCACCACAAGCATC	53/30	55/2	659	Pru p/du 2.03
15	GW4	PruTLP3-GSP1 PruTLP3-GSP2	AGTACTCACCCACCAACTTCTCCTGCGT GCTACTTTAGCCGAGTTCACTCTCAACG			800/1000	Pru p/du 2.03
16	Consensus of 29 ESTs	PpTLP4f PpTLP4r	ACTTGTCTGAACTTATGGCTCC GAAGTTAGAAGAAGAGCTGCG	58/30	60/2	1800/1825	Pru p/du 2.04
17	AY620230 X96714	PpLTP1f1 PpLTP1r	ATCATAGTCAAGAGAGATGG CCCTAAGTGGATCACATAGC	52/30	54/2	645	Pru p/du 3.01
18	AY093699 X96716	PpLTP2f PpLTP2r	TATCAGCTTTACTTACGACG CTGGCTTCCACAGAAACCTC	58/30	60/2	513/514	Pru p/du 3.02
19	X96715	PpLTP3f PpLTP3r	CCCAAGCGAAAGAAACACTA CATCTCATATCATCCTTCCA	58/30	60/2	527/528	Pru p/du 3.03
20	AJ491881 AY081852	Pp4.01f Pp4.01r	AGAAGAAATCAGAAGCAACG AAATAGTCACTCGGAGCAAT	51/30	53/2	1041	Pru p/du 4.01
21	AJ491882	Pp4.02f Pp4.02r1	CAGCAACAACAACAAAGATG TCTAGAGACCCTGCTCAACC	53/30	55/2	754	Pru p/du 4.02

^1^GW-genome walking, only gene specific primers (GSP) shown, according to sequences obtained with previous primer group, representative reference EST accessions for group 16: [GenBank:DY634569; DY644567; DY651848; DY634415]

### Genome walking

Genome walking was applied to gDNA of MB1-73 using the Universal Genome Walker kit (Clontech, Palo Alto Calif.) for gene sequences that were not cover the ORF or to avoid the mistakes induced by primer sequence, or to explore polymorphisms in the flanking regions. Four libraries were constructed using *Dra *I, *EcoR *V, *Pvu *II and *Stu *I enzymes to digest 2.5 μg of gDNA from MB1-73. Adaptors were ligated to the digested DNA fragments. Two gene specific primers (GSP, Table 4) and two adaptors were used for nest PCR. The product from two of the four libraries was excised from the gel and subsequently purified, ligated, transformed and sequenced as described in the above section.

### Sequence analysis

DNA sequences were analysed and single nucleotide polymorphisms (SNPs) were identified using the SEQMAN program (Lasergene 6, Madison, Wis.). Intron sizes were deduced by comparing the genome sequences with cDNA (ESTs) sequences or by putative conserved splicing patterns in plants. The phylogenetic tree was created and percentage sequence identity calculated using Clustal W by Megalign (Lasergene 6). The GenDoc program  was used for multiple DNA and amino acid sequence alignment.

### Allele-specific markers and bin mapping approach

Three types of molecular markers were used: 1) two dominant single nucleotide amplification polymorphism (SNAP) markers [18] representing T and E respectively, 2) co-dominant markers based on length differences due to Indels and variation in the number of simple sequence repeats (SSRs) and 3) SNP markers based on direct sequencing of the PCR products from 8 bin samples. Markers 1) and 2) were used for the mapping of *Pru p 1, 2*, and *4 *genes and 3) for *Pru p 3 *genes.

With SNAP markers, the annealing temperature is usually critical. It was optimised by gradient PCR using gDNAs of 'Texas' almond and 'Earlygold' peach (the two parents of MB1-73) as contrasting templates, and then applied to genomic DNA of 8 bin samples. Indels and SSR markers were revealed by 6% PAGE and silver staining. For direct sequencing, after PCR amplification (GoTaq, Promega), an aliquot of the PCR reaction was loaded on an agarose gel to check for specific amplification. If the PCR produced a single fragment, the remaining PCR reaction was purified using Roche High Pure PCR Product purification kit (Germany). An aliquot of 0.5 μl to 5 μl of purified PCR was used as template for sequencing with forward and/or reverse primers. SNPs and/or indels were detected using Sequencher software, version 4.7.

For mapping, we followed the strategy of "selective" or "bin" mapping [26,37] where, based on the data of a detailed map already constructed (in this case the TxE map), a few plants are selected having a high number of recombination breakpoints and an approximately even distribution of these breakpoints throughout the genome. The joint genotype of these plants for a new marker defines a single map position, allowing its mapping with less time and cost, and with a moderate loss of precision compared with the use of the whole population. For *Prunus*, a set of only eight plants: one of the parents ('Earlygold') the F1 hybrid (MB1-37) and six plants (# 5, 12, 23, 30, 34 and 83) from the TxE progeny, was selected by Howad et al. [26] allowing to establish the position of any marker to 67 fragments of the genome ("bins") of an average size of 7.8 cM. The identification of each bin is unequivocal except for two pairs (2:45/3:04 and 5:41/8:30), which have an identical genotype and require the genotype of an additional F2 plant (#27) to separate them. By comparing the genotype appeared in H and E, we were able to distinguish T allele from that of E. Marker tests were performed on these eight individuals of the bin set that were genotyped as homozygous for the 'Texas' allele (noted as A), homozygous for the 'Earlygold' allele (B) or heterozygous as MB1-73 (H). The resulting segregation pattern was compared to the defined data for each bin, and the matching bin was identified [26].

### Nomenclature of the putative allergen sequences

We followed the systematic allergen nomenclature [38] for the four different protein classes and encoding genes. Generally, allergens are designated by the first three letters of the name of the genus, followed by a space and the first one or two letters of the species name. These letters are usually followed by a space and an Arabic number according to the order in which the allergens were identified, whereby the same number is used to designate homologous allergens of related species. Additional numbers and letters can be added to distinguish different isoallergens and variants [19]. Accordingly, PR-10, TLP, LTP and PRF proteins are denoted as Pru p 1 to 4 in peach and Pru du 1 to 4 in almond. Amino acid sequences with more than 5% dissimilarity usually belong to a different isoallergen and are distinguished by a two-digit code. If there are several genes within isoallergen group (on the DNA sequence identity, usually more than 95%), they are further distinguished by adding a capital letter after their isoallergen name, such as Pru p 2.01A and Pru p 2.01B. This procedure has been employed in apple [17] and birch [39]. The plant material in this study is an interspecies hybrid between peach and almond; we assigned two alleles of the same gene locus by its original species source, from *Prunus persica *as Pru p and from *Prunus dulci *as Pru du. Therefore, *Pru p/du *refers to a gene in interspecies hybrid parent MB1-37.

## Authors' contributions

ZSG, KSC, EVW, PA and SDW initiated this study; ZSG, LC and WH designed the experiment. LC, SMZ, LJS and ZSG performed the gene cloning, sequence analysis and mapping of *Pru p/du 1*, *Pru p/du 2 *and *Pru p/du 4*. EI and WH did the bin mapping of *Pru p/du 3*; ZSG, LC, EVW, PA and KSC drafted the paper.
